# Mechanisms of Response and Resistance to Immune Checkpoint Inhibitors in Renal Cell Carcinoma

**DOI:** 10.3390/cells15211954

**Published:** 2026-10-27

**Authors:** Martina Milella, Francesco Lasorsa, Monica Rutigliano, Alessandro Caniglia, Emanuele Naglieri, Savio Domenico Pandolfo, Pasquale Ditonno, Giuseppe Lucarelli

**Affiliations:** 1 Urology Unit, IRCCS Istituto Tumori “Giovanni Paolo II”, 70124 Bari, Italy; 2 Urology and Kidney Transplantation Unit, Department of Precision and Regenerative Medicine and Ionian Area, University of Bari “Aldo Moro”, 70124 Bari, Italy; 3 Pathology Unit, IRCCS Istituto Tumori “Giovanni Paolo II”, 70124 Bari, Italy; 4 Oncology Unit, IRCCS Istituto Tumori “Giovanni Paolo II”, 70124 Bari, Italy; 5 Department of Urology, University of L’Aquila, 67100 L’Aquila, Italy; 6 Department of Neurosciences, Science of Reproduction and Odontostomatology, Federico II University, 80138 Naples, Italy

**Keywords:** renal cell carcinoma, immune checkpoint inhibitors, tumor microenvironment, immunotherapy resistance, biomarkers

## Abstract

Renal cell carcinoma (RCC), particularly its clear-cell subtype (ccRCC), is characterized by metabolic reprogramming and a complex tumor microenvironment that influence responses to immune checkpoint inhibitors (ICIs). Despite clinical advances, therapeutic resistance remains a major hurdle. Here we review the molecular determinants governing ccRCC sensitivity and resistance to ICIs, integrating insights from genomic alterations such as *PBRM1* and *BAP1* mutations, immune cell exhaustion dynamics, stromal and metabolic barriers, and the role of endogenous retroviral antigens. We discuss how these factors modulate immune evasion and impact therapeutic outcomes. Emerging strategies including biomarker-driven patient stratification, HIF-2α inhibition, and personalized immunotherapies show promise in overcoming resistance. Our synthesis highlights the necessity of multi-omics approaches to tailor immunotherapy, aiming to enhance durable responses and survival in ccRCC patients.

## 1. Introduction

Kidney malignancies are significantly represented by renal cell carcinoma (RCC), which constitutes approximately 3–5% of adult oncological diagnoses [1]. In the United States alone, recent projections by the American Cancer Society estimated an annual incidence of roughly 81,800 novel cases alongside 14,890 attributable deaths [2]. On a global scale, the clear-cell phenotype (ccRCC) remains the most frequently encountered histological subtype. Based on recent transcriptomic analyses, evidence strongly implies that these tumors histogenetically arise from proximal tubular epithelial cells (PTECs) [2,3,4]. Crucially, ccRCC is widely characterized as a metabolic disorder due to the extensive rewiring of biochemical pathways that drives its pathogenesis [5,6,7,8]. To sustain rapid, unconstrained proliferation and survival, malignant cells execute comprehensive metabolic reprogramming. Prominent among these shifts is the aberrant induction of hypoxia-inducible factor (HIF) signaling, which accelerates glucose internalization and shifts energy production toward oxygen-independent pathways [9,10,11,12,13]. Furthermore, dysregulation of lipid homeostasis is a hallmark of RCC, manifested through the heightened assimilation of fatty acids and a pronounced intracellular deposition of polyunsaturated fatty acids [14,15,16].

Although surgical intervention remains a cornerstone in managing this malignancy, approximately one-third of individuals present with metastatic disease at initial diagnosis, and roughly 25% experience disease recurrence following curative nephrectomy [17,18,19]. This clinical reality underscores the pressing requirement for innovative biomarkers that function not merely as diagnostic tools, but also as robust prognostic indicators and predictors of therapeutic efficacy [20,21,22,23]. For advanced stages, systemic regimens predominantly rely on molecularly targeted therapies, including tyrosine kinase inhibitors (TKIs) and immune checkpoint inhibitors (ICIs). Crucially, the highly heterogeneous tumor microenvironment (TME) acts as a pivotal determinant in these settings, frequently orchestrating resistance mechanisms that impair the efficacy of such immunotherapies and targeted agents [24,25]. To clarify these dynamics, this review elucidates the fundamental molecular mechanisms governing ccRCC responsiveness to immunotherapy, while comprehensively exploring the biological pathways that drive treatment resistance. The therapeutic relevance of this requirement has grown further now that checkpoint blockade has moved into the perioperative setting. In the phase III KEYNOTE-564 trial, adjuvant pembrolizumab conferred a significant overall survival advantage over placebo in resected intermediate-high-risk, high-risk or M1 no-evidence-of-disease ccRCC, and with a median follow-up of 69.5 months the benefit was sustained (5-year disease-free survival 60.9% versus 52.2%, hazard ratio [HR] 0.71; 5-year overall survival 87.7% versus 82.3%, HR 0.66) [26,27]. Because a substantial fraction of these patients would never have relapsed and are therefore exposed to immune-related toxicity without benefit, molecular stratification of both the adjuvant and the metastatic setting has become a central translational priority [28].

## 2. The Impact of the Tumor Microenvironment on RCC

Within the RCC microenvironment, tumor-infiltrating CD8+ T lymphocytes typically display an exhausted and dysfunctional state, characterized by the marked upregulation of inhibitory immune checkpoints—most notably programmed death-1 (PD-1), cytotoxic T-lymphocyte-associated protein 4 (CTLA-4), and T cell immunoglobulin and mucin domain-containing protein 3 (TIM-3)—in conjunction with a diminished expression of proliferation indicators like Ki-67 [29,30]. Intriguingly, a comprehensive meta-analysis demonstrated that intratumoral CD8+ T cell density paradoxically portends a dismal prognosis in RCC, contrasting sharply with patterns observed in most other solid malignancies [31]. Although the exact etiology of this phenomenon is still under active investigation, it is widely hypothesized that persistent exposure to an immunosuppressive milieu within the tumor stroma compromises the capacity of CD8+ T cells to recognize specific antigens, undergo clonal expansion, and produce interleukin-2 (IL-2), thereby neutralizing their cell-killing effectiveness [32]. Importantly, recent work has refined how these dysfunctional populations should be interpreted. Antigen reactivity, rather than shared residency markers, distinguishes bona fide exhausted T (TEX) cells—which experience continuous cognate antigen exposure, express high levels of coinhibitory receptors and track with responsiveness to checkpoint blockade—from tissue-resident memory (TRM) cells, which are frequently low-avidity or bystander clones spatially segregated from their antigen, retain superior cytokine output and instead correlate with improved survival after resection [33]. Since both compartments co-express CD69 and CD103 and are captured together by bulk CD8 quantification, this distinction offers a plausible explanation for why undifferentiated CD8+ density behaves as an adverse prognostic variable in RCC while functionally defined exhausted subsets predict benefit from PD-1 blockade.

At the molecular level, recent mechanistic insights reveal that sustained antigen engagement triggers the activation of nuclear factor of activated T-cells (NFAT) independently of AP-1. This isolated activation induces the transcriptional regulators TOX and WNK1, definitively directing CD8+ T lymphocytes toward a terminal exhaustion lineage [34]. Specifically in RCC, the engagement of CD8+ T-cell PD-1 by tumor-expressed PD-L1 recruits SLC11A1, which subsequently blunts ZAP70 and PI3K/AKT downstream signaling cascades; simultaneously, CTLA-4 outcompetes CD28 for B7 costimulatory molecules on antigen-presenting cells, thereby dampening essential secondary activation signals [35,36,37]. Furthermore, signaling mediated by the TIM-3–galectin-9 axis upregulates BATF transcription factor levels, reinforcing this non-responsive gene expression profile [38]. Collectively, these inhibitory pathways attenuate the release of key cytotoxic mediators, including granzyme B and interferon-gamma (IFN-γ), while severely dampening proliferative responses.

**Figure 1:**
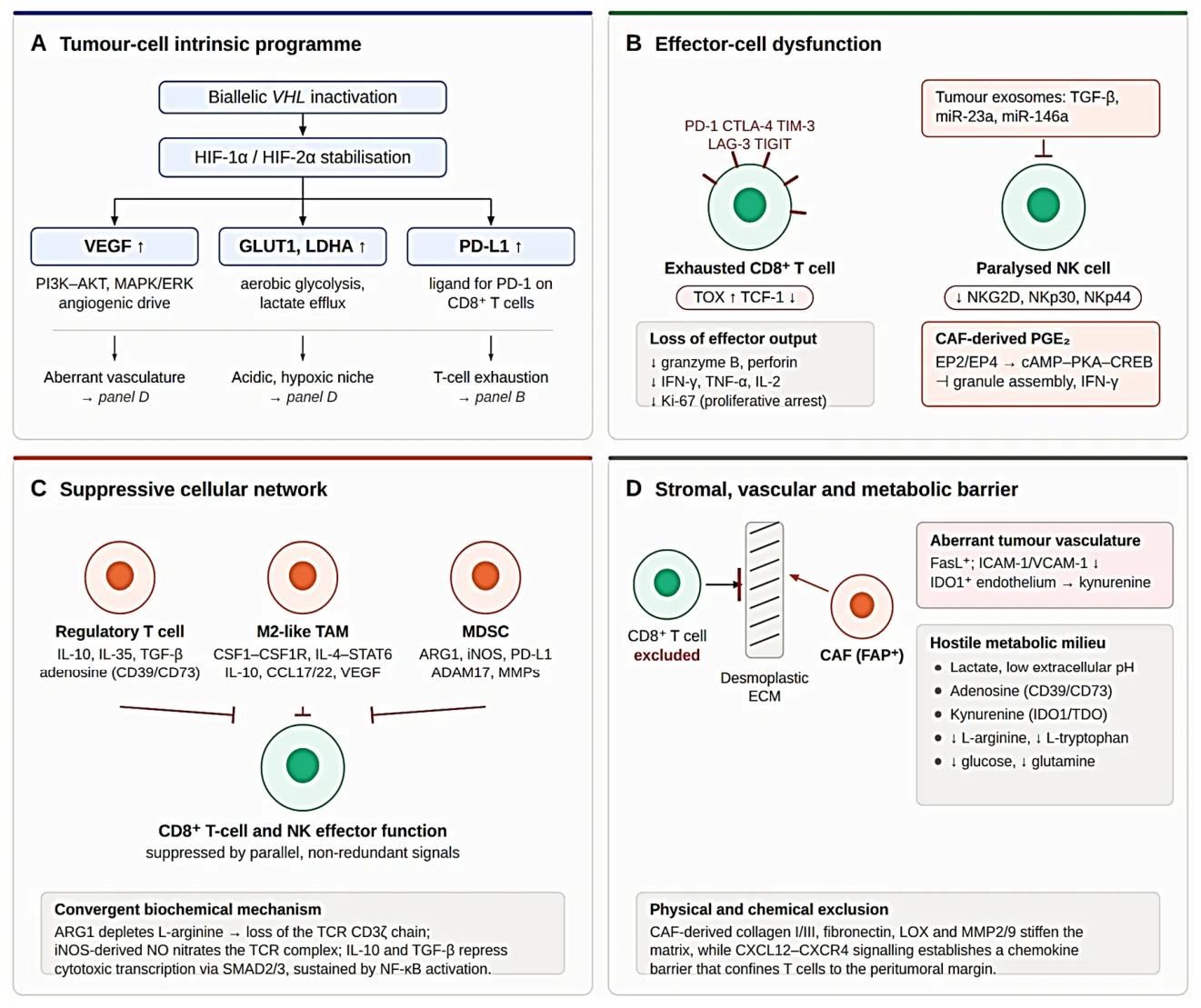
**The immunosuppressive tumor microenvironment of clear cell renal cell carcinoma.** (**A**) Tumor-cell intrinsic program. Biallelic *VHL* inactivation prevents degradation of HIF-1α and HIF-2α, whose stabilization drives three downstream branches: VEGF overexpression with PI3K–AKT and MAPK/ERK activation, generating the aberrant vasculature shown in (**D**); induction of GLUT1 and LDHA with aerobic glycolysis and lactate efflux, generating the acidic, hypoxic niche shown in (**D**); and PD-L1 upregulation, providing the ligand for PD-1 on infiltrating CD8+ T cells as shown in (**B**). (**B**) Effector-cell dysfunction. Tumor-infiltrating CD8+ T cells co-express PD-1, CTLA-4, TIM-3, LAG-3 and TIGIT with high TOX and reduced TCF-1, and lose granzyme B, perforin, IFN-γ, TNF-α, IL-2 and Ki-67 expression. NK cells are paralyzed by tumor-derived exosomes carrying TGF-β, miR-23a and miR-146a, which downregulate NKG2D, NKp30 and NKp44, and by CAF-derived PGE2 signaling through EP2/EP4 to the cAMP–PKA–CREB axis to repress granule assembly and IFN-γ synthesis. (**C**) Suppressive cellular network. Regulatory T cells (IL-10, IL-35, TGF-β, CD39/CD73-derived adenosine), M2-like tumor-associated macrophages (CSF1–CSF1R and IL-4–STAT6 polarization; IL-10, CCL17, CCL22, VEGF) and myeloid-derived suppressor cells (ARG1, iNOS, PD-L1, ADAM17, MMPs) converge on CD8+ T-cell and NK-cell effector function through parallel, non-redundant mechanisms: ARG1-mediated L-arginine depletion causes loss of the TCR CD3ζ chain, iNOS-derived nitric oxide nitrates the TCR complex, and IL-10 and TGF-β repress cytotoxic transcription Via SMAD2/3, sustained by NF-κB activation. (**D**) Stromal, vascular and metabolic barrier. FAP+ cancer-associated fibroblasts deposit collagen I/III, fibronectin, LOX and MMP2/9 that stiffen the matrix, while CXCL12–CXCR4 signaling forms a chemokine barrier confining CD8+ T cells to the peritumoral margin. Aberrant vessels express FasL, downregulate ICAM-1 and VCAM-1, and display IDO1+ endothelium generating kynurenine; the surrounding milieu is rich in lactate and adenosine and depleted of L-arginine, L-tryptophan, glucose and glutamine. Abbreviations: ARG1, arginase-1; CAF, cancer-associated fibroblast; CCL, C-C motif chemokine ligand; CREB, cAMP response element-binding protein; CSF1R, colony-stimulating factor 1 receptor; CXCL/CXCR, C-X-C motif chemokine ligand/receptor; ECM, extracellular matrix; FAP, fibroblast activation protein; GLUT1, glucose transporter 1; HIF, hypoxia-inducible factor; ICAM-1, intercellular adhesion molecule 1; IDO1, indoleamine 2,3-dioxygenase 1; iNOS, inducible nitric oxide synthase; LDHA, lactate dehydrogenase A; LOX, lysyl oxidase; MDSC, myeloid-derived suppressor cell; MMP, matrix metalloproteinase; PGE2, prostaglandin E2; PKA, protein kinase A; TAM, tumor-associated macrophage; TCR, T-cell receptor; TGF-β, transforming growth factor beta; VCAM-1, vascular cell adhesion molecule 1; VEGF, vascular endothelial growth factor.

Recent single-cell RNA sequencing (scRNA-seq) inquiries across melanoma and renal neoplasms have mapped this heterogeneity, identifying distinct subsets of dysfunctional T cells delineated by robust co-expression of PD-1, TOX, and CXCL13, which points to dedicated anatomical micro-niches supporting these cell states [39,40]. Consequently, the operational state of CD8+ T cells serves as a critical determinant governing both survival outcomes and responsiveness to immune checkpoint blockade. The strategic application of scRNA-seq technology—already proven effective in defining melanoma biology [41]—offers a powerful tool to dissect RCC-specific TME landscapes, ultimately providing a molecular rationale for the long-standing inverse correlation between CD8+ T-cell infiltration and patient survival [42] (Figure 1).

### 2.1. Natural Killer Cell Paralysis and Immunosuppressive Cell Dynamics in RCC

NK lymphocytes represent a pivotal innate cytotoxic subset that can directly initiate anti-tumor responses via perforin- and IFN-γ-dependent pathways without requiring prior antigen presentation [43]. Indeed, elevated intratumoral NK cell density has been demonstrated to correlate with improved clinical outcomes in RCC [44]. Nevertheless, within the neoplastic microenvironment, these effector cells are frequently paralyzed by a combination of soluble factors, surface ligands, and tumor- or stroma-derived exosomes enriched in transforming growth factor-beta (TGF-β) [45,46,47]. Specifically, RCC-secreted extracellular vesicles shuttle both TGF-β and immunomodulatory microRNAs—such as miR-23a and miR-146a—into NK cells, where they induce the down-regulation of key activating receptors, including NKG2D, NKp30, and NKp44, thereby compromising tumor recognition and cytolytic clearance [48,49,50].

Concurrently, cancer-associated fibroblasts (CAFs) release substantial amounts of prostaglandin E2 (PGE2), which binds to NK cell EP2 and EP4 receptors [51,52]. This interaction triggers the intracellular cAMP–PKA–CREB signaling axis, ultimately repressing the transcription of machinery essential for cytotoxic granule assembly and IFN-γ synthesis [53,54]. Consequently, NK cells exhibit reduced proliferative capacity, impaired exocytosis, and attenuated target killing—defects that act in synergy with immunosuppressive metabolites like adenosine to systematically blunt NK cell activity within the RCC stroma [55]. Mechanistically, TGF-β signals through TGF-βRII on NK cells to activate the SMAD2/3 cascade, suppressing NKG2D and perforin expression; in parallel, extracellular adenosine engages A2A receptors to activate protein kinase A (PKA), thereby destabilizing NK cell metabolism and granule release [51,52,53]. Reversing these suppressive constraints to reactivate NK cell effector function represents a promising avenue to enhance the efficacy of immune checkpoint inhibitors in RCC [54].

In addition to NK cells, regulatory T cells (Tregs)- a CD4+ subpopulation crucial for peripheral tolerance- are actively exploited to enforce immune evasion within the RCC tumor microenvironment [56]. Chemokines, cytokines (IL-10, IL-23, TGF-β), adenosine, and cell adhesion molecules produced by malignant and stromal cells orchestrate Treg recruitment. Once localized, Tregs suppress CD8+ effector T cells through TGF-β, IL-10, and IL-35 release [57]. While Treg infiltration is generally linked to dismal clinical outcomes, the exact functional nuances governing their activity in RCC remain to be fully delineated [58].

Among myeloid cells, TAMs constitute the predominant immune infiltrate in RCC, encompassing a spectrum between pro-inflammatory M1 and immunosuppressive M2 phenotypes [59]. A high prevalence of M2 TAMs—or an elevated M2/M1 ratio—consistently portends a poor prognosis. Driven primarily by CSF1/CSF1R and IL-4/STAT6 signaling axes, M2 polarization (often promoted by RCC-derived M-CSF) accounts for up to 20.9% of total tumor-infiltrating immune cells [60,61]. These M2 TAMs secrete IL-10, CCL17, CCL22, and VEGF, while concurrently activating pro-survival PI3K/AKT and STAT3 signaling pathways in tumor cells to accelerate proliferation, immune evasion, and matrix metalloproteinase (MMP)-dependent extracellular matrix (ECM) remodeling for invasion [62,63,64,65].

Furthermore, myeloid-derived suppressor cells (MDSCs) impair CD8+ T-cell function via PD-L1 expression, arginase-1 (ARG1)-mediated amino acid deprivation, and ADAM17-regulated leukocyte trafficking, while contributing to matrix turnover via MMPs and inducible nitric oxide synthase (iNOS) [66]. At the biochemical level, ARG1 depletes local L-arginine to downregulate CD3ζ chain expression, whereas iNOS-derived nitric oxide nitrates TCR complexes to disrupt signal transduction—a suppressive program sustained by NF-κB activation [67,68,69]. Finally, angiogenesis and tumor progression are further facilitated by Tie2-expressing monocytes (TEMs), as well as by neutrophil proteases that remodel the ECM through peptidylarginine deiminase 4 (PAD4)-driven chromatin decondensation, thereby promoting T-cell exclusion, exhaustion, TKI resistance, and adverse survival outcomes in RCC [70].

### 2.2. The Stromal, Vascular, and Metabolic Architecture Driving Immune Evasion in RCC

CAFs, the most abundant non-immune stromal cells in RCC, act as primary orchestrators of tumor progression, metastatic dissemination, therapeutic resistance, and immune exclusion [71,72]. Under hypoxic and oxidative stress, cancer-derived TGF-β, IL-6, and PDGF activate precursors into fibroblast activation protein (FAP)-positive CAFs [73]. Once activated, CAFs engage STAT3 and NF-κB signaling, releasing HGF, EGF, and IL-6 to recruit Tregs and suppressive myeloid subsets [74,75,76]. Furthermore, CAF-secreted TGF-β and ARG2 drive M2 macrophage polarization, while CXCL12 production establishes a chemokine barrier via CXCR4 engagement, physically excluding CD8+ T lymphocytes from tumor nests and directly blunting cytotoxic responses [77].

As the primary “architects” of the TME, CAFs deposit extensive ECM components—including collagens, laminins, fibronectin, and proteoglycans—that provide structural and biomechanical support for invasion [78,79]. Matrix-modifying enzymes such as MMP2/9, transglutaminases, and lysyl oxidase (LOX), induced by TGF-β and HIF-1α, drive collagen crosslinking, tissue stiffening, and reduced vascular permeability, establishing a dense desmoplastic shield that restricts immune infiltration and underlies the poor responsiveness to systemic therapies [80,81]. While targeting CAFs holds promise, their marked intratumoral heterogeneity necessitates RCC-specific strategies.

This fibrotic stroma intimately interacts with the RCC vasculature, which is characteristically hyperplastic due to early biallelic *VHL* inactivation [82]. Impaired pVHL-mediated degradation leads to HIF stabilization and overproduction of VEGF, triggering PI3K–AKT and MAPK/ERK pathways to drive angiogenesis [83].

However, the resulting vessels are functionally aberrant, expressing FasL while downregulating ICAM-1/VCAM-1 adhesion molecules, thereby impairing immune cell adhesion and trafficking [84]. Poor perfusion within this malformed bed fuels localized hypoxia and acidosis, triggering the release of immunosuppressive mediators like TGF-β, VEGF, and adenosine [85,86,87,88,89]. Additionally, tumor endothelial cells express indoleamine 2,3-dioxygenase 1 (IDO1) upon IFN-γ exposure, driving tryptophan catabolism into kynurenine, which engages the aryl hydrocarbon receptor (AHR) in T cells to induce FOXP3 expression and expand Tregs [90].

Crucially, these structural and vascular abnormalities converge with profound metabolic reprogramming driven by HIF signaling [91]. RCC cells preferentially utilize aerobic glycolysis, resulting in massive lactate accumulation and extracellular acidification [92]. Ambient lactate impairs CD8+ T-cell glycolytic capacity and mTOR signaling, driving metabolic exhaustion while promoting histone lactylation, which epigenetically upregulates PD-1 expression to amplify T-cell dysfunction synergistically with PD-1/PD-L1 signaling [93,94]. Concurrently, lactate accumulation favors Treg expansion and M2 macrophage polarization, creating a self-reinforcing loop of immune evasion [95,96]. These metabolic barriers intersect with severe nutrient depletion: malignant cells aggressively consume glucose and glutamine—essential for T-bet expression and CD4+ T-cell differentiation—while myeloid iNOS/ARG1 and endothelial IDO1 deplete L-arginine and tryptophan, generating toxic metabolites that directly compromise T-cell effector functions [97].

In conclusion, the distinctive immunological and metabolic features of the RCC microenvironment—including immune cell dysfunction, stromal barriers, and metabolic reprogramming—provide the foundation for the clinical development and implementation of ICI-based therapeutic strategies.

## 3. Therapeutic Rationale for ICI in Renal Cell Carcinoma

Despite the clinical efficacy of ICIs in advanced ccRCC, therapeutic responses remain heterogeneous, with durable remissions achieved only in a subset of patients [98,99].

To overcome primary and acquired resistance, contemporary immuno-oncology focuses on multi-targeted strategies that concurrently disarm checkpoint pathways and reprogram the immunosuppressive TME Via Treg depletion, M1 macrophage repolarization, cytokine modulation, and vascular normalization. At the mechanistic level, monoclonal antibodies targeting PD-1, PD-L1, and CTLA-4 disrupt the physiological brakes exploited by malignant cells to evade immune surveillance [100].

PD-1 blockade (e.g., nivolumab, pembrolizumab) prevents the receptor from engaging its ligands (PD-L1/PD-L2) on tumor and stromal cells, thereby reversing T-cell exhaustion and restoring proliferative and cytolytic capacity [101,102,103,104]. Conversely, anti-PD-L1 agents (e.g., atezolizumab, avelumab) selectively disrupt the PD-L1/PD-1 axis while preserving PD-L2 signaling, potentially conferring a distinct toxicity profile [105,106]. Operating earlier in the immune cascade within secondary lymphoid organs, CTLA-4 inhibition (e.g., ipilimumab) blocks CTLA-4 binding to CD80/CD86, thereby enhancing T-cell priming and expanding the pool of effector lymphocytes available for tumor infiltration [105].

Clinically, these agents have revolutionized treatment paradigms based on patient risk stratification [7]. For favorable-risk patients, management ranges from active surveillance and metastasectomy to frontline VEGFR-TKI monotherapy (pazopanib or sunitinib) or ICI–TKI combinations, such as pembrolizumab plus axitinib or lenvatinib, and nivolumab plus cabozantinib [107,108]. For intermediate- and poor-risk individuals, first-line care relies predominantly on dual immune checkpoint blockade with nivolumab plus ipilimumab or potent ICI–TKI doublets (e.g., pembrolizumab with axitinib or lenvatinib; nivolumab or avelumab with cabozantinib or axitinib), with cabozantinib offering broad anti-tumor activity across risk categories [109]. Long-term follow-up has since consolidated these regimens as standards of care rather than provisional options. The final analysis of CheckMate 214, at a median follow-up of 9.3 years, confirmed a durable advantage for nivolumab plus ipilimumab over sunitinib in the intention-to-treat population (median overall survival 52.7 versus 37.8 months, HR 0.71; 108-month overall survival 31.4% versus 19.5%), with a complete response rate of 12.0% versus 3.5% and a median duration of response of 76.2 versus 25.1 months—the longest such plateau yet reported in advanced RCC and a clinical signature of genuine immunological memory rather than cytostatic disease control [110]. In parallel, the 5-year analysis of KEYNOTE-426 sustained the superiority of pembrolizumab plus axitinib (median overall survival 47.2 versus 40.8 months, HR 0.84; median progression-free survival 15.7 versus 11.1 months, HR 0.69; objective response rate 60.6% versus 39.6%) [111]. Notably, intensification beyond doublet therapy has not translated into survival gain: in the final analysis of the phase III COSMIC-313 study, adding cabozantinib to nivolumab plus ipilimumab prolonged progression-free survival (16.6 versus 11.2 months) and response rate (46% versus 36%) but left overall survival unchanged (41.9 versus 42.0 months, HR 1.02) at the cost of substantially greater grade ≥3 toxicity [112]. These data reframe the therapeutic question from how much treatment to give to which biological subset of tumors requires which mechanism of intervention [28].

**Figure 2:**
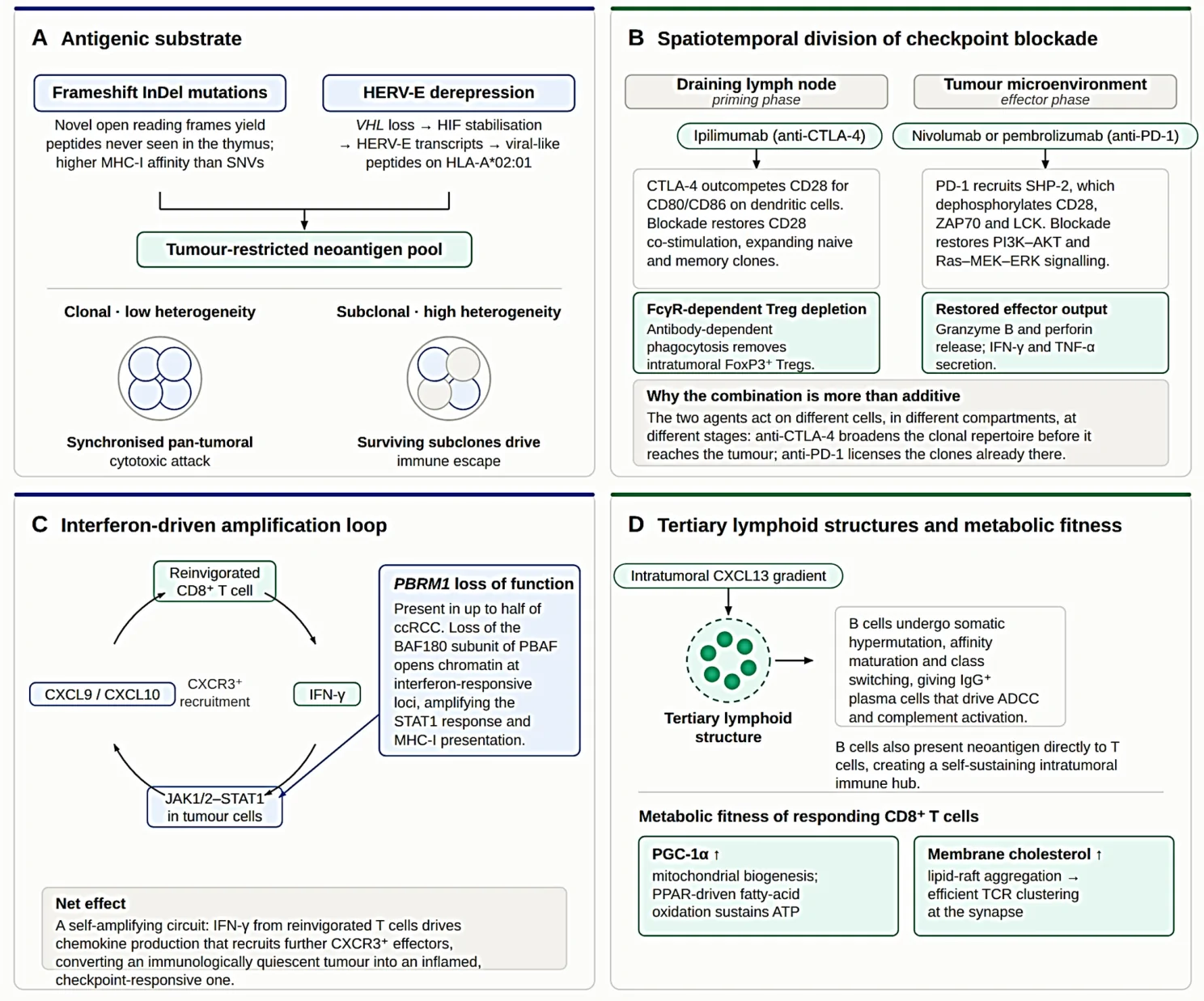
**Molecular determinants of response to immune checkpoint inhibition in ccRCC.** (**A**) Antigenic substrate. Despite a low tumor mutational burden, ccRCC generates a tumor-restricted neoantigen pool from two sources: frameshift insertion–deletion mutations, whose novel open reading frames yield peptides that escaped thymic selection and bind MHC class I with higher affinity than single-nucleotide variants; and *VHL*-loss–driven HIF stabilization, which derepresses HERV-E transcription to produce viral-like peptides presented on HLA-A*02:01. Durable responses require clonal neoantigens in tumors of low intratumoral heterogeneity, permitting synchronized pan-tumoral cytotoxicity, whereas subclonal antigens leave surviving subclones that drive immune escape. (**B**) Spatiotemporal division of checkpoint blockade. In the draining lymph node (priming phase), CTLA-4 outcompetes CD28 for CD80/CD86 on dendritic cells; ipilimumab restores CD28 co-stimulation and expands naive and memory clones and additionally depletes intratumoral FOXP3+ regulatory T cells through Fcγ receptor-dependent antibody-dependent cellular phagocytosis. Within the tumor (effector phase), PD-1 ligation recruits SHP-2, which dephosphorylates CD28, ZAP70 and LCK; nivolumab or pembrolizumab restores PI3K–AKT and Ras–MEK–ERK signaling and the release of granzyme B, perforin, IFN-γ and TNF-α. The two agents therefore act on different cells, in different compartments, at different stages. (**C**) Interferon-driven amplification loop. IFN-γ from reinvigorated CD8+ T cells activates JAK1/2–STAT1 in tumor cells, inducing CXCL9 and CXCL10, which recruit CXCR3+ effectors and close a self-amplifying circuit. Loss-of-function *PBRM1* mutation, present in up to half of ccRCC, removes the BAF180 subunit of the PBAF complex, opening chromatin at interferon-responsive loci and amplifying both the STAT1 response and MHC class I antigen presentation. (**D**) Tertiary lymphoid structures and metabolic fitness. Intratumoral CXCL13 gradients nucleate tertiary lymphoid structures in which B cells undergo somatic hypermutation, affinity maturation and class-switch recombination to yield IgG+ plasma cells driving antibody-dependent cellular cytotoxicity and complement activation, while also presenting neoantigen directly to T cells. Responding CD8+ tumor-infiltrating lymphocytes sustain effector function through PGC-1α-driven mitochondrial biogenesis with PPAR-dependent fatty-acid oxidation, and through increased membrane cholesterol promoting lipid-raft aggregation and efficient TCR clustering at the immunological synapse [146,147]. Abbreviations: ADCC, antibody-dependent cellular cytotoxicity; ccRCC, clear cell renal cell carcinoma; HERV-E, human endogenous retrovirus type E; HLA, human leukocyte antigen; InDel, insertion–deletion; MHC, major histocompatibility complex; PBAF, polybromo-associated BAF; PGC-1α, peroxisome proliferator-activated receptor gamma coactivator 1-alpha; PPAR, peroxisome proliferator-activated receptor; SNV, single-nucleotide variant; TLS, tertiary lymphoid structure.

The remarkable sensitivity of ccRCC to these immune-based strategies presents a unique biological paradigm; unlike other solid tumors, in which a high tumor mutational burden (TMB) dictates immunogenicity, ccRCC typically features a low TMB yet exhibits profound immune responsiveness [113] (Figure 2).

Mechanistically, this immunogenicity stems from a high frequency of insertion-deletion (InDel) mutations that generate frameshift neoantigens capable of eliciting potent cytotoxic T-lymphocyte responses, alongside endogenous retroviral (ERV) elements—derepressed following epigenetic dysregulation—that encode novel immunogenic peptides to fuel sustained T-cell recognition [114]. Building upon this distinct immunogenic background, the clinical success of dual checkpoint inhibition (e.g., ipilimumab plus nivolumab) relies on complementary spatiotemporal mechanisms, wherein CTLA-4 blockade acts predominantly in lymph nodes to expand polyclonal naive T-cell clones, while PD-1/PD-L1 inhibition operates peripherally within the tumor mass to reinvigorate exhausted intratumoral effectors [115]. Beyond dual checkpoint blockade, pairing ICIs with VEGFR-targeted TKIs provides target-level immunomodulatory synergy; by reversing VEGF-induced endothelial dysfunction, restoring leukocyte adhesion molecules (ICAM-1/VCAM-1), and suppressing MDSCs and Tregs, VEGFR-TKIs normalize tumor vasculature and enhance T-cell infiltration, creating a permissive microenvironment that amplifies PD-1/PD-L1 blockade efficacy [116,117]. The therapeutic sufficiency of the retroviral arm of this antigenic repertoire has likewise been shown clinically: autologous T cells engineered with a T-cell receptor recognizing a HERV-E-derived epitope mediated objective tumor regression in patients with metastatic ccRCC, establishing HERV-E as a true tumor-restricted antigen rather than an incidental transcriptional byproduct of *VHL* loss [118].

Despite these mechanistic advancements, establishing reliable predictive biomarkers for ICI response in ccRCC remains an unmet clinical need, as immunohistochemical PD-1/PD-L1 expression is constrained by spatial intratumoral heterogeneity and dynamic expression profiles [119]. Consequently, translational focus has shifted toward genomic and transcriptomic signatures, where loss-of-function *PBRM1* mutations correlate with heightened ICI sensitivity, whereas *BAP1* mutations delineate an immunosuppressive phenotype associated with poorer outcomes, and transcriptomic profiling into “angiogenic-high” versus “immuno-high” clusters aids in tailoring frontline therapy selection [120]. To further overcome treatment resistance, current research explores novel co-inhibitory receptors (such as LAG-3, TIM-3, and TIGIT co-expressed on exhausted T cells) and next-generation targeted combinations, notably the integration of HIF-2α inhibitors like belzutifan, which directly target downstream oncogenic drivers of *VHL* inactivation to dismantle both metabolic and immunological drivers of tumor progression [121]. The prospectively planned biomarker program of KEYNOTE-426 has since put this reasoning on firmer footing. In that analysis, a T-cell-inflamed gene expression profile was positively associated with objective response, progression-free survival and overall survival in the pembrolizumab plus axitinib arm but not in the sunitinib arm, whereas an angiogenesis signature predicted outcome chiefly under sunitinib—direct evidence that these signatures are treatment-specific rather than merely prognostic. Critically, PD-L1 combined positive score showed no predictive value for the immunotherapy-containing regimen, formally closing the question for this combination, while *PBRM1* mutation associated with a higher response rate to pembrolizumab plus axitinib (71.4% versus 52.3%) and *BAP1* mutation with inferior survival [111]. Complementary spatial and multiplexed work indicates that the composite of tertiary lymphoid structures together with exhausted tissue-resident T cells discriminates responders from non-responders more reliably than any single-analyte assay [122], supporting a shift toward integrated architectural rather than expression-based biomarkers.

Spatial and temporal heterogeneity pose an additional challenge to the reliability of tissue-based biomarkers. Multiregional sequencing has demonstrated that ccRCC) evolves through branched trajectories, in which “driver” events occurring after the loss of VHL—including alterations in PBRM1, SETD2, and BAP1—are often subclonal and distributed non-uniformly across different tumor regions; consequently, a single biopsy may provide an inaccurate representation of the genomic landscape and immune status of the entire tumor [123,124]. Liquid biopsy offers a potential tool for capturing this heterogeneity in an integrated and repeatable manner. Circulating tumor DNA (ctDNA) can reflect the mutational repertoire of various tumor regions; when analyzed through longitudinal sampling, it has enabled the monitoring of disease progression, in some cases even allowing for the detection of minimal residual disease or disease progression during systemic treatment well before it could be identified via radiological imaging [125,126]. However, ccRCC is a malignancy characterized by low levels of ctDNA. Untargeted plasma sequencing has detected ctDNA in only 27.5% of patients, whereas the use of personalized assays on plasma and urine samples has increased this figure to approximately 50% [125]. Approaches that do not rely on the identification of mutations may overcome this limitation; for instance, profiling the methylome of cell-free DNA in plasma has made it possible to distinguish patients with RCC from control subjects, with an area under the curve of 0.99 [127]. Furthermore, elevated levels of circulating “kidney injury molecule-1” (KIM-1) following nephrectomy have been associated with an increased risk of recurrence, suggesting its potential utility as a marker for microscopic residual disease [128]. Although none of these assays has yet been validated as a predictor of response to ICIs, integrating serial liquid biopsy with tissue-based and spatial biomarkers could mitigate sampling bias, enable dynamic monitoring of clonal evolution under therapeutic pressure, and facilitate the early identification of acquired resistance.

Ultimately, the therapeutic rationale for ICI in ccRCC relies on a delicate balance between disarming tumor-mediated immunosuppression, exploiting unique neoantigenic profiles, and capitalizing on anti-angiogenic synergy, where transitioning from empirical risk stratification to biomarker-driven personalized immunotherapy will be essential to maximize treatment efficacy while minimizing toxicity.

### 3.1. Mechanisms of Response to ICI in ccRCC

The clinical efficacy of ICIs in ccRCC relies on a unique biological architecture that sets this malignancy apart from traditional high-mutation tumors. Unlike melanoma or non-small cell lung cancer, where therapeutic sensitivity correlates directly with overall single-nucleotide variants, ccRCC generates a robust anti-tumor immune response through distinct non-synonymous mutational patterns, endogenous retroviral re-expression, chromatin remodeling events, tertiary lymphoid structure assembly, and immunometabolic adaptations.

At the genomic level, the primary antigenic substrate driving sensitivity to immune checkpoint blockade stems from insertion-deletion (InDel) mutations that induce open reading frame shifts [129]. These frameshift InDels generate novel, non-self-peptide sequences that lack central thymic tolerance and exhibit significantly higher binding affinity for MHC class I molecules compared to single nucleotide variants. Crucially, durable clinical responsiveness relies on a high clonal neoantigen load in the presence of low intratumoral heterogeneity [130]. When neoepitopes are clonal—meaning they are homogeneously expressed across all tumor subclones—T-cell receptor engagement triggers a synchronized, pan-tumoral cytotoxic attack that eliminates subclonal immune escape mechanisms. Operating alongside this mutational landscape, inactivation of the *VHL* gene leads to the stabilization of hypoxia-inducible factors HIF-1α and HIF-2α, which drives the transcriptional derepression of human endogenous retroviruses, particularly HERV-E [131]. Transcripts from HERV-E produce viral-like peptides that are presented on HLA-A*02:01, recognized by CD8+ T-cell clones as foreign antigens, thereby orchestrating dense lymphocyte infiltration into the core of the tumor microenvironment [132]. That this antigenic substrate is genuinely actionable has now been demonstrated prospectively: in a phase I study, personalized neoantigen peptide vaccines administered after nephrectomy for high-risk stage III–IV ccRCC induced vaccine-specific T-cell responses in all nine treated patients, with rapid (within 3 weeks), high-magnitude (mean 166-fold) and durable (up to 3 years) expansion of new clonotypes, reactivity against autologous tumor in seven of nine patients, and no recurrences at a median follow-up of 40.2 months [133]. Immunogenic epitopes were derived from canonical RCC drivers including *VHL*, *PBRM1*, *BAP1*, *KDM5C* and *PIK3CA*, indicating that the low-TMB, InDel-weighted mutational landscape of ccRCC nonetheless yields shared, targetable antigens.

At the cell-surface level, the PD-1 and PD-L1 signaling axis operates as a dominant operational checkpoint. Binding of PD-1 on tumor-infiltrating lymphocytes to PD-L1 on ccRCC and tumor-associated myeloid cells triggers the recruitment of the protein tyrosine phosphatase SHP-2 to the intracellular ITSM motif [35]. SHP-2 directly dephosphorylates CD28 and T-cell receptor signaling intermediates such as ZAP70 and LCK, terminating downstream PI3K-AKT and Ras-MEK-ERK signaling cascades [134]. Monoclonal antibodies directed against PD-1, such as nivolumab or pembrolizumab, or against PD-L1, such as avelumab, neutralize this inhibitory signaling. This intervention restores T-cell receptor signal transduction, unleashes lytic granule release containing granzyme B and perforin, and triggers high-level secretion of interferon-gamma and TNF-α [135]. The released interferon-gamma binds to receptors on neighboring tumor cells and vascular endothelium, activating the JAK1/2-STAT1 pathway to upregulate the expression of CXCL9 and CXCL10 chemokines. These ligands engage CXCR3 receptors on circulating effector lymphocytes and natural killer cells, generating a paracrine chemotactic gradient that amplifies immune cell trafficking into the tumor parenchyma [136].

When anti-PD-1 blockade is combined with anti-CTLA-4 therapy, such as ipilimumab, therapeutic synergism is achieved through spatiotemporal divergence in their mechanisms of action [137]. While anti-PD-1 reinvigorates exhausted effector cells locally within the tumor core, anti-CTLA-4 operates primarily within secondary lymphoid organs. CTLA-4 normally outcompetes the co-stimulatory receptor CD28 for binding to CD80 and CD86 on antigen-presenting cells. By blocking CTLA-4, ipilimumab restores CD28-mediated co-stimulation during initial antigen presentation, driving the expansion of naive and memory CD4+ and CD8+ T-cell pools [138]. Furthermore, CTLA-4 is constitutively expressed at high levels on immunosuppressive FOXP3+ regulatory T cells within the tumor bed. Anti-CTLA-4 antibodies containing functional Fc regions engage Fc-gamma receptors on intratumoral macrophages, inducing antibody-dependent cellular phagocytosis that selectively depletes regulatory T cells within the tumor core, thereby lowering the threshold for effective cytotoxic immunity [139].

Epigenetic mechanisms further modulate sensitivity to checkpoint inhibition, particularly through loss-of-function mutations in *PBRM1*, which encodes the BAF180 subunit of the PBAF SWI/SNF chromatin remodeling complex [140]. Occurring in up to half of all ccRCC cases, *PBRM1* loss alters nucleosome positioning across the genome and increases chromatin accessibility at interferon-responsive loci. Upon exposure to microenvironmental interferon-gamma, *PBRM1*-deficient ccRCC cells mount an amplified STAT1-dependent transcriptional response [141]. This leads to the hyper-secretion of T-cell recruiting chemokines and the upregulation of MHC class I antigen presentation, converting an immunologically quiescent tumor into a highly inflamed, immune-responsive microenvironment [142].

Deep and sustained responses to immunotherapy are also heavily dependent on the humoral arm of the adaptive immune system [143]. Responding tumors display a marked enrichment of B cells organized within tertiary lymphoid structures, which are ectopic lymphoid formations driven by intratumoral CXCL13 gradients [144]. Within these tertiary structures, B cells undergo somatic hypermutation, affinity maturation, and class-switch recombination, ultimately differentiating into mature IgG-producing plasma cells [145]. The locally secreted anti-tumor antibodies bind tumor-surface antigens, triggering antibody-dependent cellular cytotoxicity via natural killer cells and activating the classical complement cascade. Simultaneously, intratumoral B cells serve as efficient antigen-presenting cells that internalize neoantigens and present them to surrounding helper and cytotoxic T cells, creating a self-sustaining immune hub [145]. Direct clinical validation of this humoral axis has now been obtained in RCC: in patients treated with PD-1 blockade, the presence of mature tertiary lymphoid structures, particularly when accompanied by exhausted tissue-resident T cells in their vicinity, was associated with clinical response, whereas their absence characterized refractory tumors [122]. Tertiary lymphoid structures therefore behave less as a passive correlate of inflammation than as the anatomical site at which productive anti-tumor immunity is organized and sustained.

Finally, to maintain effector functions within the hypoxic and nutrient-deprived ccRCC microenvironment, responding CD8+ tumor-infiltrating lymphocytes undergo metabolic reprogramming [146]. Activated lymphocytes upregulate the master transcriptional coactivator PGC-1α, which drives mitochondrial biogenesis and shifts cellular energy production toward PPAR-induced fatty acid oxidation. This adaptation enables T cells to maintain high ATP output despite localized glucose starvation [147]. In parallel, increased plasma membrane cholesterol levels in immune cells promote lipid raft aggregation. This structural shift facilitates efficient T-cell receptor clustering at the immunological synapse, maximizing downstream signal transduction and lytic granule exocytosis upon neoantigen engagement.

### 3.2. Clinical Management of Cumulative Toxicity in Combination Therapies

The survival benefits achieved with combination therapeutic regimens come with a cumulative toxicity burden that has become a critical factor in the administration of treatment. In the case of dual immune checkpoint blockade, immune-related adverse events (irAEs) have been the predominant issue: in the CheckMate 214 study, grade 3–4 treatment-related adverse events occurred in 46% of patients who received nivolumab in combination with ipilimumab, leading to treatment discontinuation in 22% of cases [98]. Combinations of ICIs and tyrosine kinase inhibitors (TKIs) compound the “on-target” effects of VEGFR inhibition—such as hypertension, diarrhea, palmar-plantar erythrodysesthesia, proteinuria, fatigue, and hypothyroidism—with immune-mediated toxicities. Consequently, in the KEYNOTE-426 trial [99], grade ≥3 adverse events (of any cause) occurred in 75.8% of patients treated with pembrolizumab plus axitinib, while in the CLEAR trial, this figure reached 82.4% for patients receiving lenvatinib plus pembrolizumab; in the latter study, 68.8% of patients required a dose reduction in lenvatinib [148]. As previously discussed, the addition of a third agent in the COSMIC-313 trial further increased the incidence of grade ≥3 toxicities, without, however, improving overall survival [112]. The primary clinical challenge lies in determining the underlying cause. Diarrhea, elevated transaminases, fatigue, thyroid dysfunction, and skin toxicity can occur with both classes of drugs; however, their management differs: adverse events associated with TKIs are addressed by interrupting treatment or reducing the dosage, whereas irAEs may require the administration of systemic corticosteroids or additional immunosuppressive therapies. Such interventions carry their own morbidity and are unnecessary if the TKI is the actual cause [149]. Expert consensus therefore recommends that, in the event of overlapping low-grade adverse events, the TKI should be the first agent to be withheld. Given that axitinib has a short plasma half-life, a rapid improvement following its discontinuation points to a TKI-related cause and allows for the reintroduction of the drug at a reduced dosage; conversely, if the clinical condition persists or worsens despite the suspension of the TKI, an immune-mediated mechanism is suggested, necessitating the discontinuation of the immune checkpoint inhibitor and the initiation of corticosteroid therapy, in accordance with the guidelines for immune-related adverse events (irAEs) [149,150,151]. TKIs with a longer half-life, such as cabozantinib and lenvatinib, extend the diagnostic window and necessitate more frequent clinical and laboratory reassessments. In the event of severe hepatotoxicity or diarrhea, it is generally necessary to discontinue both drugs, rule out other potential causes, and initiate corticosteroid treatment if an immune-mediated etiology cannot be excluded [149,151].

Several practical principles follow from this. Since ICIs do not require dose adjustments, the management of toxicity relies on the temporary interruption of immunotherapy and the titration of the TKI dosage. When managed correctly, this approach allows for the maintenance of adequate exposure to both agents. A baseline assessment of thyroid, adrenal, hepatic, renal, and cardiac function—along with blood pressure and proteinuria—followed by scheduled monitoring during the first months of treatment, enables the early detection of any overlapping adverse events [150,151]. A multidisciplinary management approach—involving endocrinology, gastroenterology, hepatology, dermatology, and cardiology—combined with patient education on the prompt reporting of emerging symptoms, reduces the risk of progression to high-grade adverse events. Following the resolution of a non-life-threatening irAE, the resumption of treatment with immune checkpoint inhibitors may be considered on a case-by-case basis. Conversely, potentially life-threatening events—such as myocarditis, severe pneumonitis, or neurological toxicity—generally preclude further exposure to the drug [150,151]. Finally, the link between the gut microbiome and the risk of severe irAEs [152] suggests that efficacy and toxicity may, at least in part, be dissociable. Overall, these considerations indicate that the long-term benefit of combination therapeutic regimens depends as much on the skillful management of cumulative toxicity as it does on the choice of the regimen itself.

## 4. Molecular and Microenvironmental Drivers of Immunotherapy Resistance

### 4.1. Genomic Alterations and Oncogenic Signaling-Driven Immune Exclusion

While clinical responses to immune checkpoint blockade in clear cell renal cell carcinoma can be profound, a substantial proportion of patients exhibit intrinsic primary resistance or eventually develop acquired progression. Unraveling the molecular architecture of non-responsive tumors reveals that intrinsic resistance is tightly governed by specific somatic driver mutations and copy-number alterations that actively orchestrate an immunologically cold or immune-excluded microenvironment. A paramount genomic determinant of therapeutic failure is the loss-of-function mutation in *BAP1*, which encodes a nuclear deubiquitinating enzyme [153]. In direct contrast to the immune-sensitizing effects observed with *PBRM1* mutations, *BAP1*-deficient ccRCC tumors display an aggressive, high-grade histopathological phenotype associated with suppressed inflammatory signaling pathways [154]. Loss of BAP1 deubiquitinase activity leads to aberrant histone modifications that perturb normal chromatin accessibility, suppressing the expression of key T-cell-attracting chemokines and driving constitutive upregulation of mTORC1 signaling [155]. Consequently, *BAP1*-mutated parenchyma exhibits a profound paucity of functional CD8+ tumor-infiltrating lymphocytes, presenting as an immunologically desertified ecosystem that inherently lacks the cellular substrates required for anti-PD-1 or anti-CTLA-4 efficacy [156].

Complementing point mutations in driver genes, broad focal genomic alterations play an equally decisive role in mediating primary checkpoint resistance. Specifically, the homozygous deletion of chromosome region 9p21.3 represents one of the most robust genetic markers of therapeutic resistance across metastatic ccRCC cohorts [157]. Locus 9p21.3 encompasses the tumor suppressor genes *CDKN2A* and *CDKN2B*, which encode the cyclin-dependent kinase inhibitors p16INK4a and p15INK4b, alongside *MTAP* [158]. The loss of p16INK4a and p15INK4b drives uninhibited CDK4/6 activity, accelerating cell-cycle progression and driving tumor cell proliferation. Beyond cell-cycle deregulation, deletion of the 9p21.3 region induces a striking immune-excluded phenotype. Tumors harboring 9p21.3 alterations demonstrate a hyper-fibrotic extracellular matrix architectural frame, where dense collagen deposition driven by aberrant stromal activation physically confines tumor-infiltrating lymphocytes to the peritumoral margins, preventing functional entry into the neoplastic parenchyma and rendering checkpoint neutralization clinically ineffective [159].

### 4.2. Transcriptional Reprogramming and Terminal T-Cell Exhaustion Dynamics

**Figure 3:**
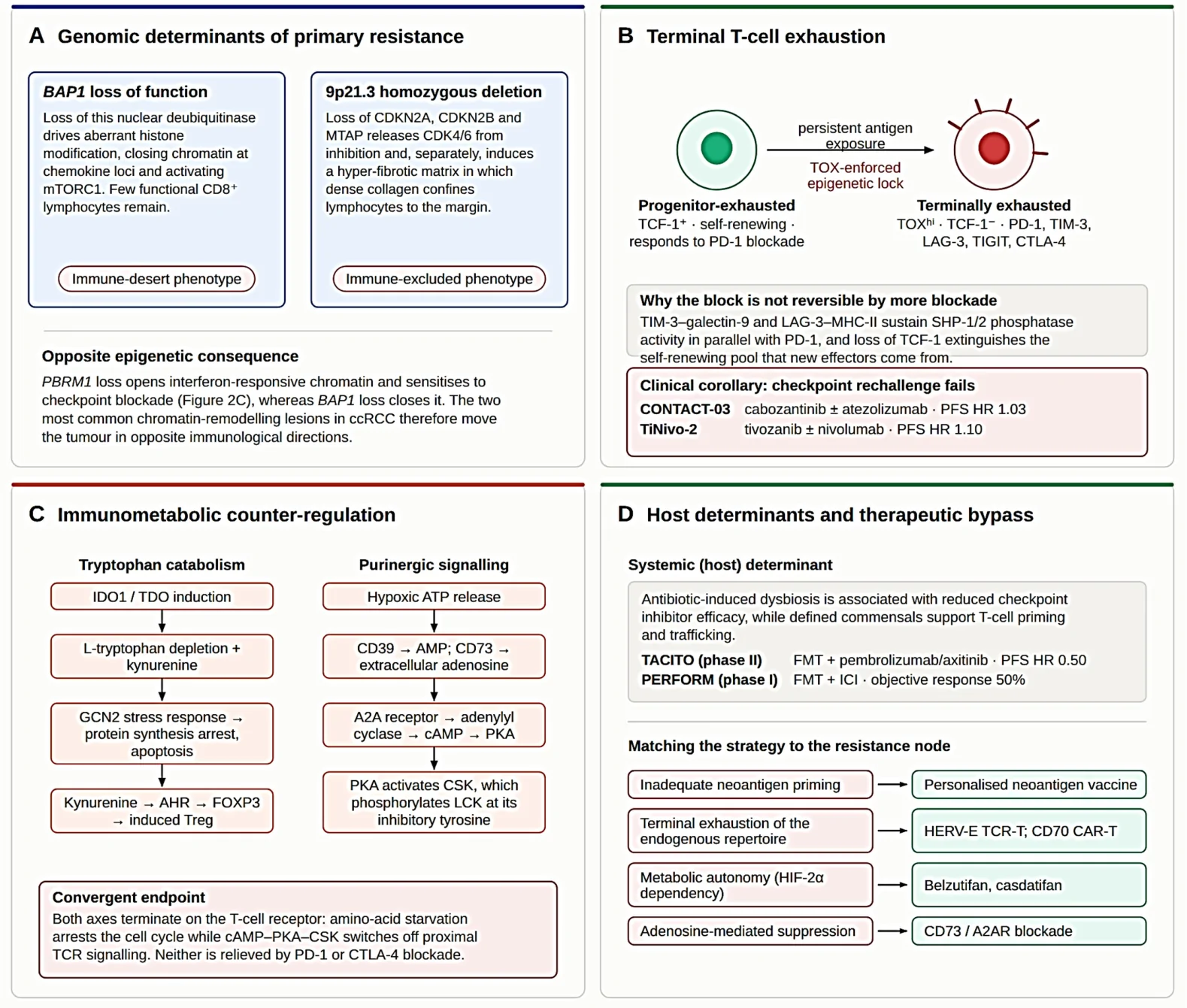
**Determinants of resistance to immune checkpoint inhibition and the strategies that bypass them.** (**A**) Genomic determinants of primary resistance. Loss of function of *BAP1*, a nuclear deubiquitinase, produces aberrant histone modification that closes chromatin at T-cell-attracting chemokine loci and activates mTORC1, yielding a high-grade, immune-desert phenotype with few functional CD8+ lymphocytes. Homozygous deletion of 9p21.3 removes *CDKN2A*, *CDKN2B* and *MTAP*, releasing CDK4/6 from inhibition and independently inducing a hyper-fibrotic matrix in which dense collagen confines lymphocytes to the peritumoral margin, giving an immune-excluded phenotype. These lesions move the tumor in the opposite immunological direction to *PBRM1* loss (Figure 2C). (**B**) Terminal T-cell exhaustion. Under persistent antigen exposure, progenitor-exhausted TCF-1+ self-renewing cells that respond to PD-1 blockade convert into a terminally exhausted, TOX-high, TCF-1-negative state co-expressing PD-1, TIM-3, LAG-3, TIGIT and CTLA-4. The block is not reversible by further blockade: TIM-3–galectin-9 and LAG-3–MHC class II engagement sustain SHP-1/2 phosphatase activity in parallel with PD-1, and loss of TCF-1 extinguishes the self-renewing pool from which new effectors arise. The clinical corollary is the failure of checkpoint inhibitor rechallenge in CONTACT-03 (PFS HR 1.03) and TiNivo-2 (PFS HR 1.10). (**C**) Immunometabolic counter-regulation. IDO1/TDO induction depletes L-tryptophan and generates kynurenine; tryptophan starvation activates GCN2, arresting protein synthesis and triggering apoptosis, while kynurenine engages the aryl hydrocarbon receptor to induce FOXP3 and convert naive CD4+ cells into regulatory T cells. In parallel, hypoxia-driven ATP release is converted by CD39 to AMP and by CD73 to extracellular adenosine, which engages A2A receptors to raise cAMP and activate PKA; PKA activates CSK, which phosphorylates LCK at its inhibitory tyrosine. Both axes terminate on the T-cell receptor and neither is relieved by PD-1 or CTLA-4 blockade. (**D**) Host determinants and therapeutic bypass. Antibiotic-induced dysbiosis is associated with reduced checkpoint inhibitor efficacy while defined commensals support T-cell priming and trafficking; fecal microbiota transplantation improved progression-free survival in the randomized phase II TACITO trial (PFS HR 0.50) and achieved a 50% objective response rate in the phase I PERFORM trial. Lower panel: emerging strategies mapped onto the specific resistance node each circumventsHERV-E-directed TCR-engineered T cells and CD70-directed CAR T cells for terminal exhaustion of the endogenous repertoire; HIF-2α inhibition with belzutifan or casdatifan for metabolic autonomy; and CD73 or A2A receptor blockade for adenosine-mediated suppression. Abbreviations: A2AR, A2A adenosine receptor; AHR, aryl hydrocarbon receptor; CAR, chimeric antigen receptor; CSK, C-terminal Src kinase; FMT, fecal microbiota transplantation; GCN2, general control nonderepressible 2; HR, hazard ratio; ICI, immune checkpoint inhibitor; PFS, progression-free survival; TCR-T, T-cell receptor-engineered T cell; TDO, tryptophan 2,3-dioxygenase; TOX, thymocyte selection-associated high mobility group box protein.

Acquired resistance, occurring in patients who experience disease progression after an initial therapeutic response, is largely dictated by the irreversible functional decay of tumor-infiltrating T lymphocytes. Under conditions of persistent, unmitigated tumor antigen exposure, antigen-specific CD8+ T cells undergo a progressive, multi-stage developmental transition from an early, progenitor-exhausted state into a terminally dysfunctional epigenetic lineage [160] (Figure 3).

The molecular switch governing this terminal commitment is centered on the high-level expression of the master transcription factor TOX (Thymocyte Selection-Associated High Mobility Group Box). TOX functions as a critical epigenetic regulator that binds directly to regulatory elements across the genome, recruiting chromatin remodeling complexes that stably alter histone methylation and acetylation patterns [161].

This TOX-enforced epigenetic architecture locks T cells into a state of permanent transcriptional repression, suppressing the expression of key cytotoxic mediators including granzyme B, perforin, interferon-gamma, and TNF-α [162]. Concurrently, TOX induces the stable, high-density co-expression of multiple inhibitory cell-surface receptors beyond PD-1, most notably TIM-3 (encoded by *HAVCR2*), LAG-3, TIGIT, and CTLA-4 [163]. When infiltrating lymphocytes reach this terminally exhausted state, monotherapy directed against the PD-1/PD-L1 axis proves insufficient to restore lytic capacity. Compensatory signaling propagated through parallel coinhibitory receptors—such as TIM-3 binding to galectin-9 or phosphatidylserine, and LAG-3 binding to MHC class II molecules—maintains robust SHP-1/2-mediated phosphatase activity, effectively bypassing PD-1 blockade and sustaining intracellular signal suppression [164]. Furthermore, terminally exhausted T cells downregulate key transcription factors necessary for memory conversion, such as TCF-1 (encoded by *TCF7*), extinguishing the self-renewing progenitor pool capable of generating fresh waves of functional effector progeny upon immune checkpoint inhibition [165]. The clinical corollary of this epigenetically fixed state has been tested directly, and the results support this interpretation.

In the phase III CONTACT-03 trial, adding atezolizumab to cabozantinib in patients progressing on or after prior checkpoint inhibition produced no improvement in progression-free survival (HR 1.03) or overall survival (HR 0.94) [166]; the phase III TiNivo-2 trial likewise found that adding nivolumab to tivozanib failed to improve progression-free survival (5.7 versus 7.4 months, HR 1.10) and concluded that checkpoint inhibitor rechallenge should be discouraged in this setting [167]. Taken together, these trials argue that once the intratumoral T-cell compartment has been driven into terminal, TOX-enforced dysfunction, re-engaging the same PD-1/PD-L1 axis cannot restore effector competence, and that meaningful salvage requires either epigenetic reprogramming of the existing repertoire, recruitment of a fresh antigen-naive repertoire, or abandonment of T-cell-dependent mechanisms altogether.

### 4.3. Myeloid-Derived Immunosuppression and Stromal Extracellular Remodeling

The cellular architecture of the non-responding ccRCC microenvironment is heavily dominated by immunosuppressive myeloid populations, particularly M2-like TAMs and Myeloid-Derived Suppressor Cells (MDSCs) [168]. Driven by high intratumoral secretion of VEGF, IL-6, and macrophage colony-stimulating factor (M-CSF), these myeloid lineages infiltrate the tumor bed and deploy diverse non-redundant biochemical mechanisms to neutralize cytotoxic T-cell activity [169]. A central pathway of myeloid inhibition is the hyper-expression of Arginase-1 (ARG1) and iNOS. These enzymes rapidly deplete the essential amino acid L-arginine from the extracellular microenvironment [170]. Extracellular L-arginine deprivation induces the loss of the CD3-zeta (CD3ζ) chain in the T-cell receptor complex on infiltrating CD8+ T cells, arresting TCR signaling, halting cell-cycle progression in the G0/G1 phase, and blunting cytokine synthesis [171]. Encouragingly, this myeloid axis appears to be pharmacologically tractable and prospectively identifiable. Exploratory biomarker analyses from COSMIC-313 showed that tumors enriched for M2-like macrophages derived disproportionate progression-free and overall survival benefit from the addition of cabozantinib to dual checkpoint blockade, whereas responders to the doublet alone were characterized by stronger immune-activation signatures and responders to the triplet by higher angiogenic signaling with lower immune activity [112]. A myeloid-high, immune-low transcriptional state may thus precisely define the subgroup in which antiangiogenic intensification, rather than further checkpoint blockade, is mechanistically warranted.

Simultaneously, M2-like TAMs and CAFs act as the primary sources of TGF-β within the ccRCC stroma. Elevated TGF-β signaling exerts a powerful dual immunosuppressive effect: it directly represses the transcription of cytotoxic gene programs in CD8+ T cells and NK cells via SMAD2/3 phosphorylation, while concurrently driving intense extracellular matrix remodeling [172]. TGF-β stimulates CAFs to synthesize dense bundles of type I and type III collagen, fibronectin, and hyaluronan, transforming the tumor stroma into a rigid, impenetrable biophysical barrier. This fibrotic wall physically sequesters cytotoxic T lymphocytes within the perivascular and stromal compartments, preventing direct physical contact between effector lymphocytes and ccRCC target cells [173]. Consequently, despite the systemic administration of anti-PD-1 or anti-CTLA-4 antibodies, the physical entrapment of immune cells within the matrix stroma establishes a state of immune exclusion that directly drives primary and secondary treatment resistance.

### 4.4. Immunometabolic Counter-Regulation and Suppressive Biochemical Axes

Beyond cellular crosstalk and structural barriers, the metabolic microenvironment of ccRCC actively enforces immune suppression through the accumulation of toxic metabolic end-products and the competitive depletion of critical nutrients. Tumor-cell metabolic adaptation creates a hostile biochemical niche that selectively impairs effector lymphocyte survival while favoring the expansion of regulatory cell populations. A prominent driver of metabolic resistance is the upregulation of the IDO1/TDO-Kynurenine signaling axis [174]. Tumor cells and infiltrating dendritic cells upregulate Indoleamine 2,3-dioxygenase 1 (IDO1) or Tryptophan 2,3-dioxygenase (TDO) in response to inflammatory cues. These enzymes catalyze the rate-limiting degradation of the essential amino acid L-tryptophan into its downstream catabolite, kynurenine [175].

The resulting local depletion of L-tryptophan activates the nutrient-sensing kinase GCN2 (general control nonderepressible 2) in infiltrating T cells, triggering an uncharged tRNA stress response that leads to immediate protein synthesis arrest, cell-cycle cessation, and apoptosis [176]. Simultaneously, the accumulated extracellular kynurenine binds directly to the cytosolic transcription factor Aryl Hydrocarbon Receptor (AHR) in naive CD4+ T cells. AHR activation induces nuclear translocation and drives the transcription of FOXP3, converting naive CD4+ helper T cells into immunosuppressive CD4+ FOXP3+ Tregs [177]. These induced Tregs further secrete IL-10 and TGF-β, establishing a self-reinforcing immunosuppressive circuit that blunts local effector T-cell proliferation and function.

Parallel to tryptophan catabolism, ectopic purine metabolism driven by the ectonucleotidase enzymatic cascade constitutes a major metabolic barrier to effective immunotherapy [178]. Under hypoxic conditions induced by *VHL* loss and tissue necrosis, ccRCC cells and tumor-associated stromal elements release substantial quantities of ATP. The sequential activity of two surface-bound ectonucleotidases—CD39 (ectonucleoside triphosphate diphosphohydrolase-1), which converts extracellular ATP to AMP, and CD73 (5′-nucleotidase), which hydrolyzes AMP—leads to the continuous accumulation of extracellular adenosine within the tumor core [179].

Extracellular adenosine binds with high affinity to A2A adenosine receptors (A2AR) selectively expressed on the surface of cytotoxic CD8+ T cells and NK cells. Engagement of A2AR activates intracellular adenylyl cyclase, causing a rapid spike in intracellular cAMP levels [180]. Elevated cAMP activates PKA, which directly phosphorylates C-terminal Src kinase (CSK). Activated CSK subsequently phosphorylates LCK at its inhibitory tyrosine residue, turning off downstream TCR signaling cascades, halting lytic granule exocytosis, and repressing the transcription of IL-2 and IFN-γ [181]. Concurrently, adenosine binding to A2B receptors on myeloid precursors promotes their differentiation into M2-like immunosuppressive macrophages, effectively shielding ccRCC cells from checkpoint-mediated immune destruction through a multi-layered biochemical barrier [182]. Beyond these suppressive circuits, the degree to which a tumor remains addicted to the pseudohypoxic program itself now appears to stratify immunological from metabolic dependency. In a translational analysis accompanying the ARC-20 study of the next-generation HIF-2α inhibitor casdatifan, tumors with high HIF-2α protein expression, elevated baseline EPO mRNA and pronounced on-treatment reduction in serum erythropoietin showed superior response and progression-free survival, whereas a HIF-2α expression signature predicted worse progression-free survival with sunitinib or immunotherapy-based combinations; progressing tumors were instead enriched for oxidative phosphorylation programs [183]. This inverse relationship implies that HIF-2α-dependent and immune-dependent ccRCC represent partially non-overlapping biological states, and that the failure of checkpoint blockade in the former reflects metabolic autonomy rather than immune exhaustion alone.

### 4.5. Host and Systemic Determinants of Checkpoint Inhibitor Sensitivity

A third axis of resistance, operating entirely outside the tumor bed, has moved from correlative observation to interventional evidence. The intestinal microbiota is increasingly regarded as a systemic determinant of checkpoint inhibitor sensitivity, with antibiotic-induced dysbiosis associated with diminished efficacy and specific commensal taxa promoting T-cell priming and trafficking [184]. In the randomized phase II TACITO trial, fecal microbiota transplantation from responder donors added to pembrolizumab plus axitinib narrowly missed its primary endpoint of 12-month progression-free survival (70% versus 41%, *p* = 0.053) but produced a marked improvement in median progression-free survival (24.0 versus 9.0 months, HR 0.50) and objective response rate (52% versus 32%) [185]. Mechanistically, benefit tracked not with global donor engraftment but with the acquisition of particular taxa—*Blautia wexlerae* associating with improved outcome and *Akkermansia massiliensis* with inferior progression-free survival—while loss of baseline *Ruminococcus bromii* predicted diminished benefit. The phase I PERFORM trial independently established the feasibility and safety of this approach, reporting a 50% objective response rate with no serious transplantation-related toxicity, and further linked durable engraftment of anti-inflammatory taxa such as *Faecalibacterium prausnitzii* to protection from severe immune-related adverse events, whereas expansion of *Segatella copri* accompanied grade ≥3 toxicity [152]. These findings position the microbiome as a modifiable determinant that acts upstream of the intratumoral events discussed above and suggest that efficacy and toxicity may be at least partially dissociable through targeted ecological manipulation.

## 5. Translating Mechanistic Insight into the Evolving Clinical Landscape

**Figure 4:**
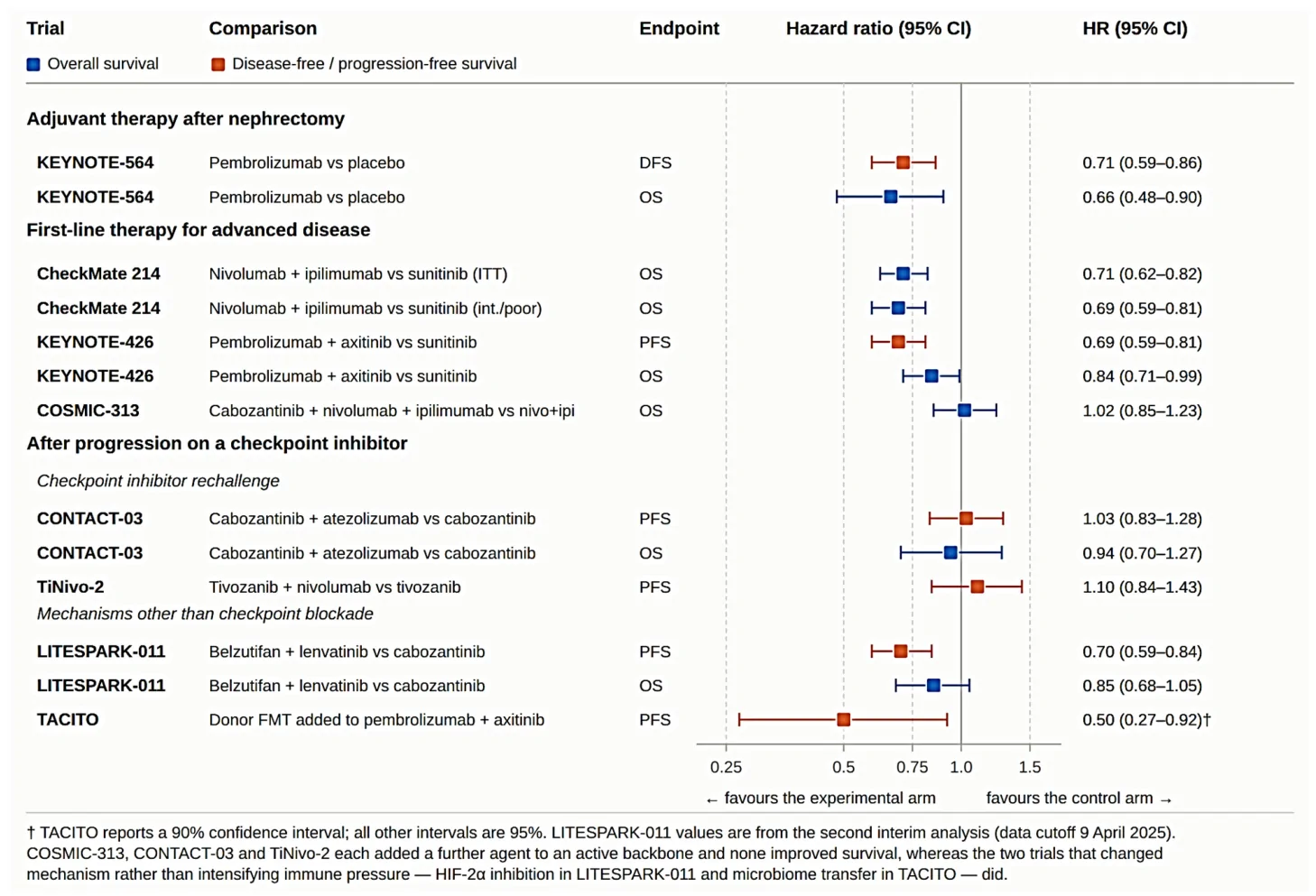
**Hazard ratios for efficacy endpoints across the checkpoint-inhibitor development program in renal cell carcinoma.** Point estimates (squares) with confidence intervals (horizontal bars) are plotted on a logarithmic scale; the vertical reference line marks a hazard ratio of 1.0. Color distinguishes overall survival from disease-free or progression-free survival, and the endpoint is also stated for each row. Trials are grouped by clinical setting: adjuvant therapy after nephrectomy; first-line therapy for advanced disease; and therapy after progression on a checkpoint inhibitor, subdivided into checkpoint inhibitor rechallenge and mechanisms other than checkpoint blockade. Checkpoint blockade confers a durable benefit in the adjuvant and first-line settings, but intensification beyond doublet therapy (COSMIC-313) and re-exposure to the PD-1/PD-L1 axis after progression (CONTACT-03, TiNivo-2) yield no survival gain, whereas benefit is restored by changing mechanism rather than intensifying immune pressure—HIF-2α inhibition in LITESPARK-011 and microbiome transfer in TACITO. † TACITO reports a 90% confidence interval; all other intervals are 95%. LITESPARK-011 values are from the second interim analysis (data cutoff 9 April 2025). Abbreviations: DFS, disease-free survival; FMT, fecal microbiota transplantation; ITT, intention to treat; OS, overall survival; PFS, progression-free survival.

The mechanisms outlined above are no longer purely descriptive: each now maps onto a distinct and clinically tested therapeutic strategy, and the pattern of successes and failures across recent phase III trials is itself mechanistically informative (Figure 4).

Three lessons emerge with particular clarity. First, the setting in which checkpoint blockade is deployed matters as much as the agent. In the adjuvant context—where micrometastatic disease presumably harbors a less established immunosuppressive stroma and a T-cell repertoire not yet driven to terminal exhaustion—pembrolizumab achieved a durable disease-free and overall survival benefit that remains evident at five years [26,27], and updated European Association of Urology guidance now incorporates adjuvant checkpoint inhibition for selected high-risk resected ccRCC [28]. Conversely, once resistance has emerged in the metastatic setting, re-exposure to the same axis is futile, as CONTACT-03 and TiNivo-2 both demonstrated [166,167]. The therapeutic window for PD-1/PD-L1 blockade is therefore bounded not by drug exposure but by the epigenetic state of the available T-cell repertoire.

Second, targeting the pseudohypoxic core of ccRCC biology provides a genuinely orthogonal mechanism of action that remains effective after immunological failure. Belzutifan established proof of principle in the phase III LITESPARK-005 study, improving progression-free survival and objective response rate (21.9% versus 3.5%) over everolimus in heavily pretreated disease, albeit without a significant overall survival gain [186]. The phase III LITESPARK-011 trial subsequently showed that belzutifan plus lenvatinib outperformed cabozantinib after anti-PD-(L)1 therapy (median progression-free survival 14.8 versus 10.7 months, HR 0.70; objective response rate 52.6% versus 40.2%; median duration of response 23.0 versus 12.3 months), with overall survival favoring the combination but not yet statistically significant [187]. Next-generation HIF-2α inhibition with casdatifan has produced durable activity in refractory disease together with pharmacodynamic and transcriptional biomarkers of HIF-2α dependency, offering for the first time a prospective means of selecting patients for this axis [183]. Because HIF-2α-dependent tumors appear to fare worse on immunotherapy combinations, these biomarkers may ultimately serve to triage patients between immunological and metabolic strategies rather than merely to sequence them.

Third, where the endogenous T-cell repertoire is exhausted or the relevant antigens are inadequately presented, supplying immunity from outside the tumor becomes the rational alternative. Personalized neoantigen vaccination generated durable, broad and tumor-reactive T-cell responses in resected high-risk ccRCC [133]; HERV-E-directed T-cell receptor gene therapy mediated objective regressions in metastatic disease [118]; and allogeneic CD70-directed chimeric antigen receptor T cells produced responses in the phase I TRAVERSE trial (objective response rate 17.4% overall, rising to 31.3% among patients with a CD70 tumor proportion score ≥50%), with responses confined to CD70-expressing tumors and dependent on adequate CAR T-cell expansion [188]. Each of these modalities bypasses a specific resistance node identified in the preceding sections—absent neoantigen priming, defective antigen presentation, and terminal exhaustion of the endogenous repertoire, respectively—and each comes with an intrinsic, measurable selection criterion. Alongside microbiome-directed intervention [152,185], these approaches sketch a plausible route from the current one-size-fits-all combination paradigm toward mechanism-matched therapy.

## 6. Conclusions and Future Perspectives

In conclusion, the therapeutic landscape of ccRCC has been radically redefined by ICIs, yet primary and acquired resistance remain formidable clinical hurdles. Understanding resistance to ICI-based regimens requires moving beyond simplistic single-biomarker models and embracing the multi-layered complexity of the ccRCC ecosystem. As highlighted throughout this review, susceptibility and refractory behavior to immunotherapy are governed by a dynamic interplay between intrinsic genomic alterations, a heterogeneous cellular microenvironment, and distinct metabolic counter-regulatory mechanisms.

Genomically, ccRCC presents a unique paradigm where high immunogenicity exists independently of a high TMB. Instead, frameshift InDels, HERVs expression, and loss-of-function mutations in chromatin remodeling genes—most notably *PBRM1*, *BAP1*, and *SETD2*—shape the baseline immune architecture and dictate sensitivity to anti-PD-1 and anti-CTLA-4 agents. However, genomic features alone cannot fully predict clinical outcomes due to the profound influence of the tumor microenvironment (TME). The infiltration of terminally exhausted CD8+ T cells expressing multiple co-inhibitory receptors (e.g., TIM-3, LAG-3), coupled with the dense recruitment of immunosuppressive M2-like TAMs, Tregs, and CAFs, creates both a physical and immunological barrier that restricts effective anti-tumor responses.

Furthermore, metabolic reprogramming driven by loss of *VHL* and tissue hypoxia acts as a powerful biochemical shield against immune destruction.

Looking toward the future, overcoming ICI resistance in ccRCC will necessitate a transition from empirical combination strategies to rational, biomarker-driven precision immuno-oncology. Current standard-of-care regimens—combining dual checkpoint blockade (e.g., nivolumab plus ipilimumab) or ICIs with anti-angiogenic TKIs (e.g., pembrolizumab plus lenvatinib or axitinib)—have significantly improved survival, largely by simultaneously targeting tumor angiogenesis and immune suppression, and their long-term follow-up now documents a genuine plateau of durable benefit in a minority of patients [110,111]. Equally instructive are the negative results: the absence of an overall survival gain from triplet intensification in COSMIC-313 [112] and the failure of checkpoint inhibitor rechallenge in CONTACT-03 and TiNivo-2 [166,167] together indicate that adding immune pressure to an already refractory microenvironment is not a viable strategy. However, next-generation therapeutic avenues must aim higher by directly targeting the metabolic and microenvironmental drivers of resistance.

**Figure 5:**
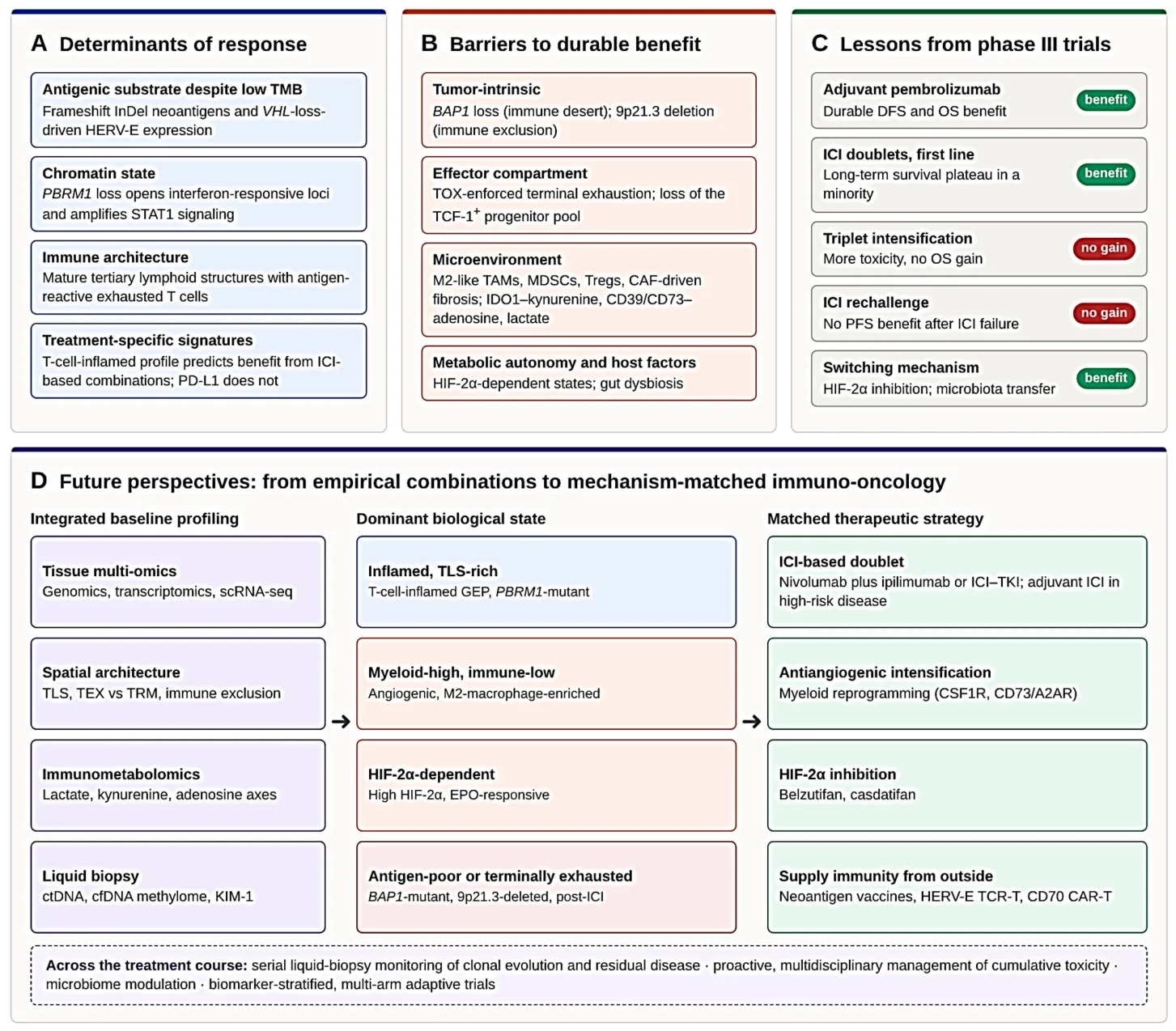
**Conclusions and future perspectives.** (**A**) Determinants of response. Despite a low tumor mutational burden, ccRCC presents an antigenic substrate derived from frameshift InDel neoantigens and *VHL*-loss-driven HERV-E expression; *PBRM1* loss amplifies interferon signaling; mature tertiary lymphoid structures containing antigen-reactive exhausted T cells organize productive immunity; and T-cell-inflamed gene expression profiles predict benefit in a treatment-specific manner, whereas PD-L1 expression does not. (**B**) Barriers to durable benefit: tumor-intrinsic lesions (*BAP1* loss, 9p21.3 deletion), TOX-enforced terminal T-cell exhaustion, a suppressive myeloid, stromal and immunometabolic microenvironment, HIF-2α-dependent metabolic autonomy and host factors such as gut dysbiosis. (**C**) Lessons from phase III trials: checkpoint blockade confers durable benefit in the adjuvant and first-line settings, whereas triplet intensification and checkpoint inhibitor rechallenge yield no survival gain, and benefit after progression is restored by switching mechanism. (**D**) Future perspectives. Integrated baseline profiling—tissue multi-omics, spatial architecture, immunometabolomics and liquid biopsy—assigns each tumor to a dominant biological state, which is matched to the strategy that addresses its limiting mechanism. Serial liquid biopsy, proactive management of cumulative toxicity, microbiome modulation and biomarker-stratified adaptive trials apply across the treatment course. Abbreviations: CAR-T, chimeric antigen receptor T cell; cfDNA, cell-free DNA; ctDNA, circulating tumor DNA; DFS, disease-free survival; EPO, erythropoietin; GEP, gene expression profile; ICI, immune checkpoint inhibitor; KIM-1, kidney injury molecule-1; OS, overall survival; PFS, progression-free survival; TCR-T, T-cell receptor-engineered T cell; TEX, exhausted T cell; TKI, tyrosine kinase inhibitor; TLS, tertiary lymphoid structure; TRM, tissue-resident memory T cell.

Ultimately, unlocking the full potential of immunotherapy in ccRCC will depend on integrated multi-omics profiling—combining spatial transcriptomics, single-cell RNA sequencing, and immunometabolomics, complemented by serial liquid biopsy—to stratify patients accurately at baseline. By matching patient-specific immune and metabolic resistance profiles with tailored multi-target therapeutic combinations, the field can move closer to durable complete responses and long-term survival for all patients with advanced renal cell carcinoma. The elements of such a strategy are now individually validated rather than hypothetical: treatment-specific transcriptional signatures and tertiary lymphoid structure architecture to identify immunologically tractable tumors [111,122]; pharmacodynamic and transcriptional markers of HIF-2α dependency to direct patients toward metabolic rather than immunological intervention [183,187]; myeloid-high, immune-low states to select those who benefit from antiangiogenic intensification [112]; antigen-directed and cell-based therapies for tumors whose endogenous repertoire is irrecoverably exhausted [118,133,188]; and microbiome modulation as a systemic, potentially toxicity-sparing adjunct [152,185]. The principal challenge ahead is no longer the identification of candidate mechanisms but their prospective integration into biomarker-stratified trial designs capable of testing several mechanism-matched hypotheses in parallel (Figure 5).

## Data Availability

No new data were created or analyzed in this study. Data sharing is not applicable to this article.

## Abbreviations

The following abbreviations are used in this manuscript:

A2AR/A2BR	A2A/A2B adenosine receptor
ADAM17	a disintegrin and metalloproteinase 17
ADCC	antibody-dependent cellular cytotoxicity
AHR	aryl hydrocarbon receptor
AKT	protein kinase B
AMP	adenosine monophosphate
AP-1	activator protein 1
ARG1/ARG2	arginase-1/arginase-2
ATP	adenosine triphosphate
B7	B7 costimulatory molecules (CD80/CD86)
BAF180	BRG1-associated factor 180 (product of *PBRM1*)
BAP1	BRCA1-associated protein 1
BATF	basic leucine zipper ATF-like transcription factor
CAF	cancer-associated fibroblast
cAMP	cyclic adenosine monophosphate
cfDNA	cell-free DNA
CAR	chimeric antigen receptor
CCL17/CCL22	C-C motif chemokine ligand 17/22
ccRCC	clear cell renal cell carcinoma
CD3ζ	CD3 zeta chain
CD39	ectonucleoside triphosphate diphosphohydrolase-1
CD73	ecto-5′-nucleotidase
CDK4/6	cyclin-dependent kinase 4/6
CDKN2A/CDKN2B	cyclin-dependent kinase inhibitor 2A/2B
CREB	cAMP response element-binding protein
CSF1/CSF1R	colony-stimulating factor 1/its receptor
CSK	C-terminal Src kinase
CTLA-4	cytotoxic T-lymphocyte-associated protein 4
ctDNA	circulating tumor DNA
CXCL9/10/12/13	C-X-C motif chemokine ligand 9, 10, 12, 13
CXCR3/CXCR4	C-X-C motif chemokine receptor 3/4
DFS	disease-free survival
ECM	extracellular matrix
EGF	epidermal growth factor
EP2/EP4	prostaglandin E receptor 2/4
EPO	erythropoietin
ERK	extracellular signal-regulated kinase
ERV	endogenous retrovirus
FAP	fibroblast activation protein
FasL	Fas ligand
FcγR	Fc gamma receptor
FMT	fecal microbiota transplantation
FOXP3	forkhead box P3
GCN2	general control nonderepressible 2
GLUT1	glucose transporter 1
HERV-E	human endogenous retrovirus type E
HGF	hepatocyte growth factor
HIF-1α/HIF-2α	hypoxia-inducible factor 1-alpha/2-alpha
HLA	human leukocyte antigen
HR	hazard ratio
ICAM-1	intercellular adhesion molecule 1
ICI	immune checkpoint inhibitor
IDO1	indoleamine 2,3-dioxygenase 1
IFN-γ	interferon gamma
IgG	immunoglobulin G
IL-2/4/6/10/23/35	interleukin-2, -4, -6, -10, -23, -35
irAE	immune-related adverse event
InDel	insertion–deletion
iNOS	inducible nitric oxide synthase
ITSM	immunoreceptor tyrosine-based switch motif
ITT	intention to treat
JAK1/2	Janus kinase 1/2
KDM5C	lysine demethylase 5C
KIM-1	kidney injury molecule-1
Ki-67	marker of proliferation Ki-67
LAG-3	lymphocyte-activation gene 3
LCK	lymphocyte-specific protein tyrosine kinase
LDHA	lactate dehydrogenase A
LOX	lysyl oxidase
M-CSF	macrophage colony-stimulating factor
MAPK	mitogen-activated protein kinase
MDSC	myeloid-derived suppressor cell
MEK	mitogen-activated protein kinase
MHC	major histocompatibility complex
miR-23a/miR-146a	microRNA-23a/-146a
MMP	matrix metalloproteinase
mRNA	messenger RNA
MTAP	methylthioadenosine phosphorylase
mTOR/mTORC1	mechanistic target of rapamycin (complex 1)
NFAT	nuclear factor of activated T cells
NF-κB	nuclear factor kappa B
NK	natural killer
NKG2D	natural killer group 2 member D
NKp30/NKp44	natural cytotoxicity receptors 30/44
NO	nitric oxide
OS	overall survival
p15INK4b/p16INK4a	cyclin-dependent kinase inhibitor proteins
PAD4	peptidylarginine deiminase 4
PBAF	polybromo-associated BAF
PBRM1	polybromo 1
PD-1	programmed cell death protein 1
PD-L1/PD-L2	programmed death-ligand 1/2
PDGF	platelet-derived growth factor
PFS	progression-free survival
PGC-1α	PPAR gamma coactivator 1-alpha
PGE2	prostaglandin E2
PI3K	phosphoinositide 3-kinase
PIK3CA	PI3K catalytic subunit alpha
PKA	protein kinase A
PPAR	peroxisome proliferator-activated receptor
PTEC	proximal tubular epithelial cell
RCC	renal cell carcinoma
scRNA-seq	single-cell RNA sequencing
SETD2	SET domain containing 2
SHP-1/SHP-2	Src homology region 2 domain-containing phosphatase 1/2
SLC11A1	solute carrier family 11 member 1
SMAD2/3	SMAD family member 2/3
SNV	single-nucleotide variant
STAT1/STAT3/STAT6	signal transducer and activator of transcription 1, 3, 6
SWI/SNF	switch/sucrose non-fermentable
TAM	tumor-associated macrophage
TCF-1 (*TCF7*)	T-cell factor 1
TCR	T-cell receptor
TDO	tryptophan 2,3-dioxygenase
TEM	Tie2-expressing monocyte
TEX	exhausted T cell
TGF-β	transforming growth factor beta
TGF-βRII	TGF-β receptor II
TIGIT	T-cell immunoreceptor with Ig and ITIM domains
TIM-3 (*HAVCR2*)	T-cell immunoglobulin and mucin domain-containing protein 3
TKI	tyrosine kinase inhibitor
TLS	tertiary lymphoid structure
TMB	tumor mutational burden
TME	tumor microenvironment
TNF-α	tumor necrosis factor alpha
TOX	thymocyte selection-associated high mobility group box protein
Treg	regulatory T cell
TRM	tissue-resident memory T cell
tRNA	transfer RNA
VCAM-1	vascular cell adhesion molecule 1
VEGF/VEGFR	vascular endothelial growth factor/its receptor
VHL	von Hippel–Lindau
WNK1	WNK lysine deficient protein kinase 1
ZAP70	zeta-chain-associated protein kinase 70
